# MET Expression and Cancer Stem Cell Networks Impact Outcome in High-Grade Serous Ovarian Cancer

**DOI:** 10.3390/genes12050742

**Published:** 2021-05-14

**Authors:** Maria Bååth, Jenny-Maria Jönsson, Sofia Westbom Fremer, Laura Martín de la Fuente, Lena Tran, Susanne Malander, Päivi Kannisto, Anna Måsbäck, Gabriella Honeth, Ingrid Hedenfalk

**Affiliations:** 1 Division of Oncology, Department of Clinical Sciences Lund, Lund University and Skåne University Hospital, 223 81 Lund, Sweden; maria.baath@med.lu.se (M.B.); jenny-maria.jonsson@med.lu.se (J.-M.J.); sofia.westbom_fremer@med.lu.se (S.W.F.); laura.martin_de_la_fuente@med.lu.se (L.M.d.l.F.); Lena.Tran@med.lu.se (L.T.); Susanne.Malander@med.lu.se (S.M.); Gabriella.Honeth@med.lu.se (G.H.); 2 Department of Surgical Pathology, Division of Laboratory Medicine, Skåne University Hospital, 222 42 Lund, Sweden; Anna.Masback@skane.se; 3 Division of Obstetrics and Gynaecology, Department of Clinical Sciences Lund, Lund University and Skåne University Hospital, 222 42 Lund, Sweden; Paivi.Kannisto@med.lu.se

**Keywords:** HGSC, cancer stem cells, SOX2, MET, OCT4, ovarian cancer, PARP

## Abstract

Overexpression of the receptor tyrosine kinase MET has been linked to poor survival in several cancer types, and MET has been suggested to interact with stem cell networks. In vitro studies have further suggested a possible benefit of a combined treatment using PARP and MET inhibitors. We used a tissue microarray (TMA) with 130 samples of advanced-stage high-grade serous fallopian tube/ovarian cancer (HGSC) to investigate the prognostic value of MET protein expression alone and in combination with the stem cell factor SOX2. The possible synergistic effects of a PARP and MET inhibitor treatment were evaluated in two cell lines with *BRCA1* or *BRCA2* deficiency and in their *BRCA1/2*-proficient counterparts. Patients with tumors positive for MET had worse overall survival (log-rank test, *p* = 0.015) compared to patients with MET-negative tumors. The prognostic role of MET was even more prominent in the subgroup of patients with SOX2-negative tumors (*p* = 0.0081). No synergistic effects of the combined treatment with PARP and MET inhibitors were found in the cell lines examined. We conclude that MET expression could be used as a marker for OS in HGSC and that stemness should be taken into consideration when evaluating the mechanisms of this effect.

## 1. Introduction

In 2020, 314,000 women worldwide were diagnosed with ovarian or fallopian tube cancer. The disease accounted for 3.4% of all female cancer cases and 4.7% of all cancer-related deaths in women [1]. There are several different histological subtypes of ovarian/fallopian tube cancer, of which high-grade serous cancer (HGSC) is the most common. Even with relatively good initial response rates of around 70% to primary surgery and chemotherapy, it is estimated that around 80% of HGSC patients will relapse; the 5-year survival is below 50% [2]. The standard first-line treatment for advanced disease is platinum in combination with a taxane. The indications for use of PARP inhibitors (PARPi) as a maintenance treatment are rapidly evolving. Current Swedish treatment guidelines stipulate that a PARPi may be used as maintenance treatment for stage III or higher disease, provided that at least a partial response is achieved and that a *BRCA1/2* mutation is present (either germline or somatic) [3]. In recurrent, platinum-sensitive disease, PARPi maintenance treatment is approved for use regardless of the *BRCA1/2* mutation status, again requiring at least a partial response to re-treatment with platinum [4,5,6]. To date, PARPi is, however, not used in platinum-resistant disease in clinical routine.

The response to PARPi is facilitated through binding of the drug to PARP proteins, thereby disrupting the repair of single-strand breaks. This in turn leads to an accumulation of double-strand breaks in cells with a defective homologous repair (HR) machinery and eventually results in cell death. HR deficiency is most commonly caused by mutations in important DNA repair genes such as *BRCA1* and *BRCA2*, but PARPi also show effect in ovarian cancer patients without *BRCA1/2* mutations. Overall survival (OS) rates have been shown to increase in both *BRCA1/2*-mutant and -wildtype carriers following PARPi treatment, provided that the tumors are still platinum-sensitive [5,6,7].

Nevertheless, many patients develop resistance to PARPi [8], and this treatment resistance can have multiple explanations. As described by Lord et al., it can be due to secondary mutations in *BRCA1/2*, loss of PARP1 expression, or increased expression of ATP-binding cassette (ABC) transporters, effectively pumping the PARPi out of the cells. Another previously investigated mechanism involves tumors overexpressing the tyrosine kinase receptor MET, which is a common feature in many cancer forms [9]. High MET expression has been linked to shorter OS in ovarian cancer [10], triple-negative breast cancer (TNBC) [11], and non-small-cell lung cancer patients [12].

Overcoming treatment resistance in an era of targeted treatments is an unmet clinical need. Several studies report that MET can phosphorylate PARP1, thereby preventing binding of the inhibitor and thus making cells PARPi-resistant [13,14]. The combination of a MET inhibitor (METi) and a PARPi has been shown to synergistically inhibit growth in TNBC cell lines [15] as well as in ovarian cancer cell lines [15,16,17].

Another possible mechanism underlying the development of resistance and relapse is that tumors may harbor cancer stem cells capable of evading chemotherapy treatment [18]. An increased population of cells positive for stem cell markers was reported after completion of chemotherapy treatment in HGSC patients [19], which could indicate resistance mechanism/s in this sub-population of cells. As cancer stem cells proliferate less rapidly and also present with other features which could help them evade cell death, including self-renewal, they may be responsible for the regrowth of the tumor even after a patient is considered to have a complete response [18]. Many different stem cell markers are continuously tested for predictive potential in survival outcome. We have previously shown an association between the expression of the embryonic transcription factor SOX2 and worse survival outcome in HGSC patients with tumor tissue remaining after primary debulking surgery [20]. Likewise, reports on the stem cell-associated transcription factor OCT4 have shown that OCT4 overexpression resulted in worse OS for patients with rectal cancer [21] and worse progression-free survival (PFS) for patients with gastric cancer [22].

Interestingly, connecting these two possible resistance mechanisms, overexpression of MET has been linked to increased levels of the stem cell markers ALDH1A3 and CD133 in breast cancer [23]. Nozaki et al. reported a correlation between MET and stem cell factor expression and survival benefit for patients with ALDH1A3-negative/MET-negative or CD133-negative/MET-negative tumors compared to patients whose tumors were positive for both markers. Bellio et al. observed an increase in *SOX2* and *POU5F1* (encoding OCT4) mRNA levels, an enrichment of ALDH-, CD133-, and CD117-positive cells, and a significant increase in sphere-forming capacity following PARPi treatment of ovarian cancer cell lines [24]. Finally, Li et al. found that expression of stem cell factors and neurosphere formation markedly increased with MET activation using hepatocyte growth factor (HGF) in glioblastoma neurospheres and, conversely, decreased when cells were treated with a METi [25].

The aim of this study was to assess whether MET overexpression or co-expression of MET and SOX2 showed any prognostic potential in a consecutive and well-annotated contemporary cohort of advanced HGSC. Based on the links between MET and cancer stem cells in multiple tumor types and the potential role of MET in treatment resistance, we also wanted to investigate the role of stem cell factors in the proposed synergistic effect of METi and PARPi in HGSC cell lines with mutations in either *BRCA1* or *BRCA2* and in their counterparts with restored *BRCA1/2* function to emulate platinum-resistant disease. The purpose was to explore if the capacity to activate a cancer stem cell network might be a clue to the ability of *BRCA1/2*-proficient cells to evade PARPi treatment.

## 2. Materials and Methods

### 2.1. Patient Samples and Tissue Microarray (TMA) Construction (Cohort 1)

Ethical approval for this study was granted by the Lund University ethics committee (Sweden), waiving the requirement for informed consent (EPN 2014/717). A consecutive cohort of 156 HGSC cases, Cohort 1, was collected at the Department of Obstetrics and Gynecology at Skåne University Hospital (Sweden) between 2011 and 2015. All cases were reviewed by a gynecologic pathologist according to the World Health Organization Classification 2014 [26] and staged according to the International Federation of Gynecology and Obstetrics [27]. After evaluation, 141 HGSC patients remained. Using formalin-fixed paraffin-embedded tissue collected from these patients, a TMA was constructed as described earlier [20,28], with an average of 6 cores per patient. When available, samples were taken from both primary and metastatic tissue. Eleven of the patients included in the TMA were in stage I–II and therefore excluded from this study, leaving 130 advanced-stage HGSC cases. Clinical data on patient characteristics as well as treatment regimens and outcome were collected.

### 2.2. Immunohistochemistry

TMA sections, 3 µm in thickness, were stained with a primary antibody anti-c-Met (clone SP44, Ventana Medical system) using the fully automated DISCOVERY ULTRA (Ventana). Antigen retrieval was performed using CC1 (pH 8.5, Ventana) for 64 min at 95 °C, and the slides were incubated with ready-to-use primary antibody for 32 min at 37 °C. The TMA was scored using PathXL (Philips) by two independent examiners (MB and LT). In case of discordance, a consensus for each core was reached through joint reevaluation and, when needed, a trained gynecologic pathologist was consulted (AM). For each patient, a mean of the cores was calculated, and expression of MET was grouped as membranous staining in 0%, <5%, 5–9%, 10–49%, or 50–100% positive cells, with either weak or moderate intensity (representative images of intensity levels are shown in Supplementary Figure S1). A cut-off of ≥5% positive cells was used for survival analyses, independently of intensity. Scoring of SOX2 expression in the same TMA was performed and described earlier [19].

### 2.3. TCGA Data

mRNA data from 489 HGSC patients included in the 2009 TCGA study [29] was retrieved from cBioPortal. Out of these, 28 were filtered out because of missing data or low-stage disease, and subsequently, 461 patients with advanced-stage HGSC were included. Correlations between MET and stem cell factors were investigated, as well as survival outcome differences based on MET expression alone or in combination with stem cell factor expression.

### 2.4. Copy Number Analyses (Cohort 2)

An additional, partially overlapping cohort of 25 HGSC patients was used for global copy number analyses (referred to as Cohort 2). This cohort was collected between 2015 and 2016 and consists of patients presenting with relapsed high-grade serous ovarian, tubal, or peritoneal carcinomas who had at least partial response to second-line platinum treatment. Patients were screened for *BRCA1/2* mutations (germline or somatic), and those positive received olaparib treatment in line with clinical guidelines at the time. Pathological *BRCA1* or *BRCA2* mutations were found in 10/25 patients. Ethical approval for this cohort was granted by the Lund University ethics committee (EPN 2016/508). Written informed consent was obtained from patients receiving the olaparib treatment, while the consent requirement was waived for the remaining participants.

For this cohort, DNA was extracted from FFPE tumor tissue using the Qiagen Allprep kit for FFPE tissue (Qiagen). Sections of the tissue were stained for validation of tumor content. SNP array analysis was performed at Eurofins Genomics Europe Genotyping A/S. The OncoScan^®^ FFPE Assay Kit (Affymetrix) was used with 80 ng DNA as input. Segmentation, calculation of ploidy, normal cell contamination, and allele-specific copy number analyses were performed using ASCAT [30] (package version 2.5.2). We defined genes or regions as amplified if they had a copy number 2× the specific ploidy of the sample and as deleted if they displayed a total copy number of 0. Gain and loss were defined as >ploidy + 0.6 and <ploidy − 0.6, respectively. An HR deficiency (HRD) score was calculated from the output of ASCAT using implementations in R as described by Telli et al. [31]. A panel of 40 HR-associated genes was used to evaluate correlations between copy numbers in HR-associated genes and HRD scores [32].

### 2.5. Cell Culture

Four HGSC cell lines were cultured. The PEO1 and PEO4 cell lines are derived from ascites from a patient with poorly differentiated serous adenocarcinoma. PEO1 was collected at first relapse when the tumor was considered platinum-sensitive, and PEO4 was collected after the second relapse, when the tumor no longer responded to platinum [33]. A *BRCA2* mutation has been detected in both lines; however, PEO4 cells also harbor a reversal mutation, restoring *BRCA2* function. PEO1 and PEO4 cell lines (ECACC) were cultured in RPMI-1640 HyClone with 10% FBS, 2 mM glutamine, 2 mM sodium pyruvate, and 1% penicillin–streptomycin (all purchased from Nordic Biolabs, Täby, Sweden). The UWB1.289 cell line is derived from an ovarian tumor of papillary serous histology with a *BRCA1* mutation and defective DNA repair. UWB1.289+BRCA1 cells are from the same line, but in this version, a *BRCA1*-containing vector was stably transfected. UWB1.289 and UWB1.289+BRCA1 (ATCC) were cultured in 50% MEGM (MEGM Bullet Kit, Lonza, Basel, Schweiz) and 50% RPMI-1640 HyClone (Nordic Biolabs). Media were supplemented with 3% FBS, and for UWB1.289+BRCA1, 200 ug/mL G-418 (Corning, Corning, NY, USA) was additionally added. Mycoplasma testing and cell line authentication for all four lines was performed by GATC (Eurofins, Luxembourg, Luxembourg).

### 2.6. In Vitro Cytotoxicity Screening

Cells were seeded in 96-well plates, and single-drug treatment was performed with a range of concentrations for carboplatin, olaparib, and crizotinib, all purchased from Selleckchem. For combination treatment assays with olaparib and crizotinib, several doses for each drug were used, and the concentrations were chosen specifically for each cell line, ranging between approximately IC5 and IC60, as determined from the single-drug treatment growth assays. Cells were incubated with drugs for 6 days, fixed with 17% *w*/*v* trichloroacetic acid (Sigma Aldrich, Saint Louis, MO, USA), and air-dried. Cells were stained with 0.4% (*m*/*v*) sulforhodamine B (Sigma Aldrich), dissolved in 1% acetic acid for 30 min, and subsequently washed with 1% acetic acid. Plates were air-dried, and the dye was dissolved in 10 mM TRIS Base (Thermo Scientific, Waltham, CA, USA) before the absorbance was measured.

### 2.7. Cancer Cell Line Encyclopedia (CCLE) Data

Copy number and mRNA expression data for the cell lines PEO1 and PEO4 were retrieved from CCLE (DepMap, Broad (2020), 21Q1 Public). Data had been log2-transformed using a pseudo-count of 1. The panel of 40 HR-associated genes described earlier [32] was used to evaluate HR function in the cell lines. A difference between the two lines was defined as >10% higher expression (log2) or copy number (log2) compared to the other cell line. Levels of mRNA expression and copy numbers of genes involved in MET signaling and stem cell pathways were also investigated.

### 2.8. Western Blot Analysis

UWB1.289 and UWB1.289+BRCA1 cells were collected by scraping on ice, and Western blot analyses were performed using TGX stain-free gels (Bio-Rad Laboratories). Following blocking in 5% milk on a shaker at 4 °C overnight, membranes were incubated with primary antibodies against SOX2 (D6D9, Cell Signaling), Nanog (CL5810, Atlas Antibodies), OCT4 (NB100-2379, Novus Biologicals, Abingdon, UK), RAD51 (14B4, Abcam, Cambridge, UK), BRCA1 (#9010, Cell Signaling, Danvers, MA, USA), and Met (D1C2, Cell Signaling) on a shaker overnight at 4 °C. The primary antibody HSP90 (68/HSP90, BD Biosciences, San Jose, CA, USA), used to ensure equal protein loading on gels, was applied to the membranes for 2 h at room temperature. The membranes were washed and incubated with HRP-conjugated secondary anti-rabbit or anti-mouse antibodies (Thermo Fisher Scientific) for 1 h at room temperature. Detection was performed with Luminata Forte Western HRD Substrate (Merck-Millipore, Burlington, MA, USA) or SuperSignal West Femto Maximum Sensitivity Substrate (Thermo Scientific, Waltham, CA, USA), using Bio-Rad’s Stain-Free imaging technology (Bio-Rad, Hercules, CA, USA). Quantification of Western blot results was performed using ImageLab^®^ (Bio-Rad), and intensity was normalized to stain-free blots.

### 2.9. Statistical Analyses

To analyze the difference in the mean of continuous variables, Student’s *t*-test alternatively linear regression was used, as indicated in the Results section. For ordinal, non-parametric data (e.g., MET expression in relation to residual disease, treatment outcome, and platinum sensitivity) Wilcoxon rank–sum test was used, and for non-ordinal variables (e.g., MET in relation to *BRCA1/2* mutation, stage, and chemotherapy regimen) Fisher’s exact test was used. To evaluate survival outcome, Cox regression was used both in a univariate model comparing MET-positive/MET-negative patients, and in a multivariate model where also age, stage, and residual tumor left after surgery were included. Age was used as a continuous variable, and stage and residual tumor as binary variables (III/IV and yes/no). Spearman rank correlation (rho) was used to evaluate co-expression of MET and SOX2 at different levels of expression. For Cox regression, when including an interaction term for MET and SOX2, MET-negative and SOX2-negative were marked as reference. All calculations, visualizations, and statistical analyses were performed using R statistical environment version 4.0.5 [34].

## 3. Results

### 3.1. MET Expression Correlates with Adverse Clinico-Pathological Variables

To determine the prognostic value of MET protein expression, we scored a TMA containing 130 advanced-stage HGSC cases by immunohistochemistry (Cohort 1). MET-positive staining (defined as ≥5% cells with membrane staining) was found for 31 out of 130 patients. The groups of patients with MET-positive vs. MET-negative tumors were comparable in age, *BRCA1/2* mutation status, and amount of tumor remaining after debulking surgery (Table 1). Patients with MET-positive tumors were, however, more often diagnosed at stage IV than at stage III and were more likely to have a poor response to chemotherapy treatment, with complete response for 60% patients with MET-positive tumors vs. 76% for patients with MET-negative tumors.

### 3.2. MET Expression Correlates with Shorter Survival, Alone and in Combination with SOX2

**Figure 1:**
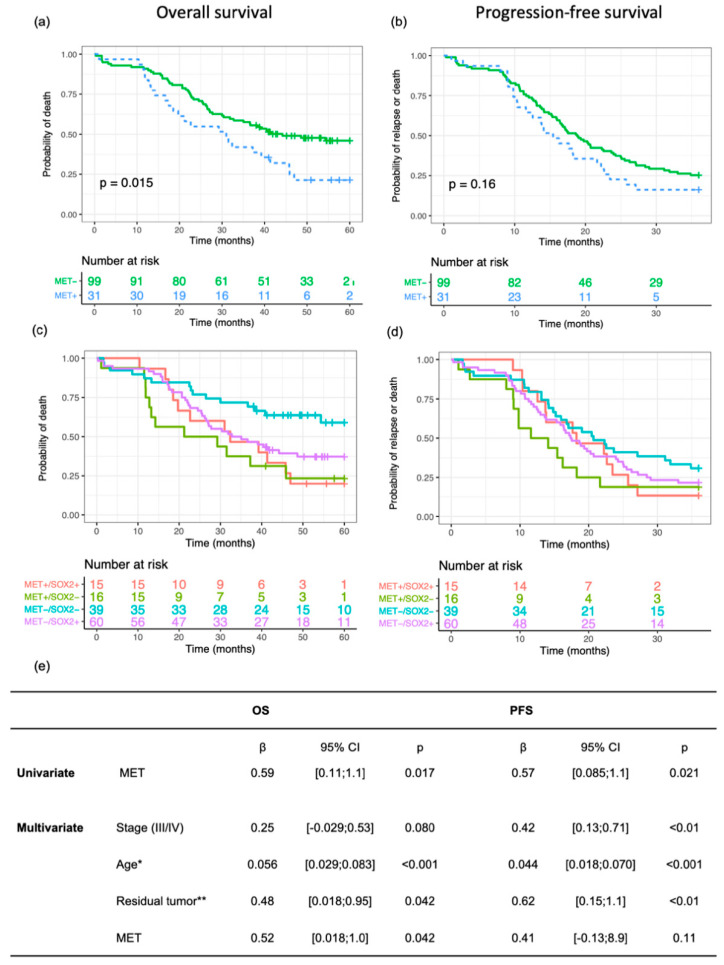
MET and SOX2 expression in relation to survival. OS and PFS for MET independently with a cut-off of ≥5% positive cells (**a**,**b**). OS and PFS in relation to co-expression of MET and SOX2 (**c**,**d**). Cox regression, univariate for MET and multivariate including MET and clinically established covariates (**e**). * Age as a continuous variable; ** Residual disease as a binary variable, with the reference set to no macroscopic tumor after primary surgery.

Kaplan–Meier analyses of OS (months from initial diagnosis to death or last follow-up) and PFS (months from the initial diagnosis to relapse or last follow-up) revealed shorter survival for patients with MET-positive tumors compared to those with MET-negative tumors (Figure 1a,b and Supplementary Figure S2). Similar results were observed in multivariate analyses. We previously assessed SOX2 expression in the described TMA cohort [19]. There was no correlation between the different levels of staining of the two markers (Spearman’s rank correlation, rho = −0.04, *p* = 0.62). When combining data for MET and SOX2 expression, we found that the subgroup of patients with tumors negative for SOX2 expression separated from those positive for SOX2. In the SOX2-negative subgroup, patients with MET-positive tumors had a significantly worse outcome than those negative for MET (log-rank *p* = 0.0081, Figure 1c,d), implying that the impact of MET expression on survival outcome is dependent on the occurrence of SOX2-positive cells in the tumors. Furthermore, Cox regression with the covariates age, stage, residual disease, and an interaction term for MET and SOX2 showed a strong and independent interaction effect between MET and SOX2 (β = −1.58, 95%CI:[−2.6;−0.56], *p* = 0.0025), indicating the significant impact of these markers on outcome. There was no significant difference in MET expression between primary and metastatic tissue (data available for 116/130 patients; mean pairwise difference 0.23%, paired *t*-test *p* = 0.84).

### 3.3. In Silico Validation Using TCGA Data

To further investigate the role of SOX2 and other stem cell markers in relation to MET expression, we retrieved a TCGA data set consisting of 489 patients, 461 of whom had both clinical and mRNA data available [28]. We evaluated the prognostic role of *MET* mRNA expression alone and in combination with four closely connected transcription factors, described as stem cell markers (*SOX2, POU5F1, Nanog,* and *KLF4*) using a cut-off of z-score > 0 to define the expression of the individual markers. None of these markers were independently connected to OS (Supplementary Figure S3), but when analyzing the subgroup of tumors negative for *POU5F1*, *MET* expression was found to be strongly prognostic, with a significantly worse outcome for patients with *MET*-positive tumors (Cox regression β = 0.5177, 95%CI:[0.19;0.85], *p* = 0.0020, Supplementary Figure S3). This effect was not seen for the remaining three transcription factors. However, when combining the four factors and analyzing the tumors lacking at least three out of four factors, *MET*-positive cases also displayed worse OS (β = 0.38, 95%CI:[0.020;0.73], *p* = 0.038).

### 3.4. HRD Scores in Relation to Copy Number Profiles, BRCA1/2 Status, and Outcome

HRD scores were calculated in 25 HGSC tumors with available copy number data (Cohort 2). HRD scores in this selected cohort ranged from 26 to 85, with a median of 57. Clinico-pathological information is shown in Supplementary Table S1. Total copy numbers relative to sample-specific ploidy in a panel of 40 HR-associated genes [31] were analyzed in relation to HRD scores, and the results are shown in Supplementary Figure S4. No general copy number differences for these genes in relation to HRD score were found. We also evaluated the genome-wide copy number profiles. Frequency plots of amplification/deletion and gain/loss in the full cohort of 25 samples, as well as for the *BRCA1/2*-mutated and -wildtype samples, separately, are shown in Supplementary Figure S5. Copy number profiles were similar in the two groups. Next, PFS to first relapse (before administration of olaparib) was compared between the *BRCA1/2-*mutant and -wildtype groups, but no significant difference was found (Cox regression coef.: −0.50, 95%CI:[−1.7;0.67], *p* = 0.39). OS was longer for patients with a *BRCA1/2* mutation, however, not to a significant degree (Cox regression coef.: −1.1, 95%CI:[−2.3;0.019], *p* = 0.070, Supplementary Table S1). All patients in the *BRCA1/2*-mutated group, but none in the *BRCA1/2*-wildtype group, received olaparib treatment at first or later relapse, as these were the clinical guidelines at the time. HRD scores were somewhat higher in the *BRCA1/2*-mutated group (median 64 for *BRCA1/2*-mutated and 52 for *BRCA1/2*-wildtype), however, not to a significant degree (Student’s *t*-test *p* = 0.10).

### 3.5. SOX2 Gene Amplification Is Correlated to a High HRD Score

**Figure 2:**
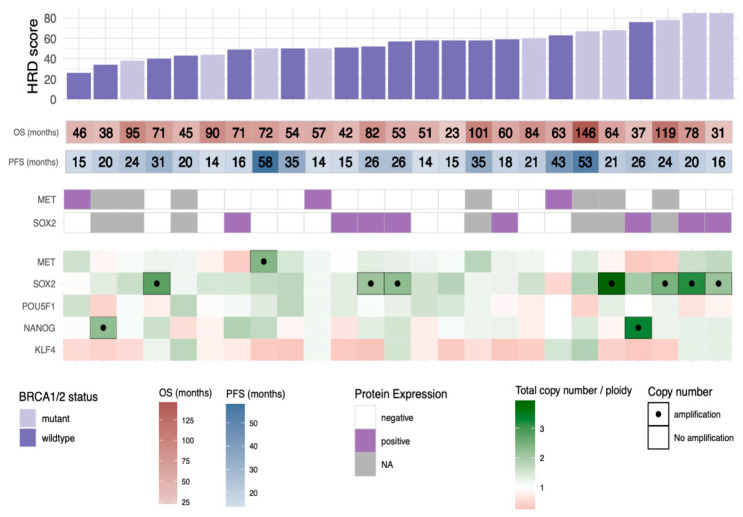
MET and cancer stem cell factors in relation to survival and HRD scores. HRD scores calculated from SNP array-based copy number data (**top** panel). MET and SOX2 expression determined by IHC in relation to OS and PFS (**middle** panel). Copy numbers of MET and the four stem cell markers relative to tumor ploidy (**lower** panel).

Eighteen patients overlapped between the two cohorts. We, hence, had both protein expression levels for MET and SOX2 and global copy number data for these patients. Relative copy number data for selected stem cell factors and protein expression for MET and SOX2, *BRCA1/2* status, HRD scores, and survival outcomes are displayed in Figure 2. SOX2 protein expression (defined as >0% positive cells detected in any core) occurred in 8/18 patients, and gene amplification in 7/18 patients. There was a positive correlation between the relative copy number of *SOX2* and HRD score (linear regression analysis, β = 9.5, *p* = 0.020, 95%CI:[1.6;17]). *MET* amplification and expression were rare in this cohort (see Figure 2), preventing evaluation of correlation with HRD scores.

### 3.6. Increased Sensitivity to PARPi In Vitro after the Development of Platinum Resistance

**Figure 3:**
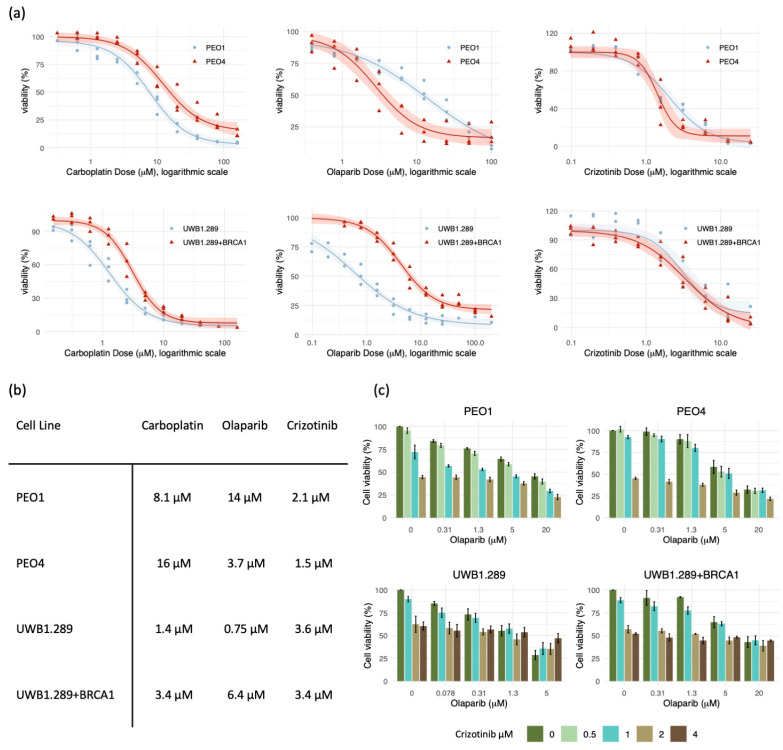
Single-drug and combination treatment of paired HGSC cell lines. Comparisons of growth inhibition within cell line pairs (**a**) and calculated IC50 values (**b**) for 6 days single-agent treatment with carboplatin, olaparib, and crizotinib. Combination treatments with olaparib and crizotinib, 6 days of incubation (**c**). No clear synergistic effects of the drug combination were detected in these cell lines.

Four HGSC cell lines were examined for drug sensitivity using platinum (carboplatin), a PARPi (olaparib), and a METi (crizotinib). The cell lines PEO1 and PEO4 were obtained from the same patient before and after platinum resistance was established. UWB1.289 and UWB1.289+BRCA1 originate from the same patient, and UWB1.289 is the parental cell line from which the UWB1.289+BRCA1 line was generated upon stable transfection with a plasmid containing a full-length functional *BRCA1* gene. These models were used for pair-wise comparisons. Differences in sensitivity within each pair for each drug are displayed in Figure 3a, and IC50 values are shown in Figure 3b. Combination treatments with olaparib and crizotinib showed no clear synergistic effects in any of the cell lines (Figure 3c).

### 3.7. PEO1 Cells Display Higher mRNA Levels of HR Genes and Cancer Stem Cell Factors Than PEO4

**Figure 4:**
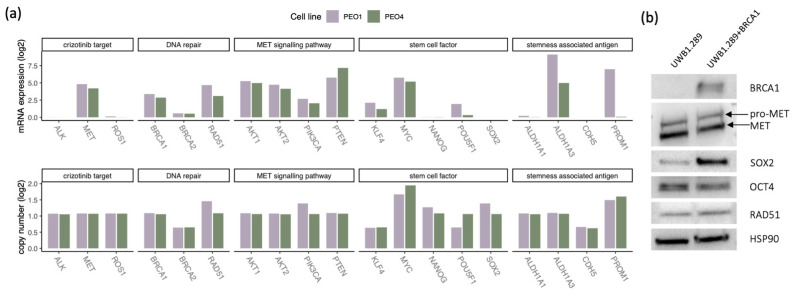
Comparing mRNA/protein expression and copy number levels within cell line pairs. mRNA expression and copy numbers for selected genes in the PEO1 and PEO4 cell lines (**a**). Protein expression levels for UWB1.289 and UWB1.289+BRCA1 cells (**b**). mRNA and copy number data were retrieved from DepMap (DepMap, Broad (2020): DepMap 21Q1 Public).

Copy number and mRNA expression data for PEO1 and PEO4, available from the CCLE database, were examined for a panel of 40 HR-associated genes [31] as well as for stem cell factors and proteins in the MET signaling pathway, allowing for in silico comparisons between *BRCA2*-deficient PEO1 and *BRCA2*-proficient PEO4 cells. By investigating the expression of HR-associated core genes, we found that these genes in general exhibited higher expression in the *BRCA2*-mutant PEO1 cell line compared to PEO4. For 20 genes, expression was higher in PEO1 cells, and for 6 genes, expression was higher in PEO4 cells. Copy numbers, however, were comparable between the cell lines. mRNA expression levels differed markedly between the cell lines for the stem cell factor *POU5F1* (encoding OCT4) as well as the stemness-associated genes *ALDH1A3* and *PROM1* (encoding CD133), with higher levels in PEO1 than in PEO4 cells. Copy number and mRNA data for stem cell factors, as well as the crizotinib targets *ALK*, *MET,* and *ROS1*, DNA repair markers *RAD51* and *BRCA1/2,* and genes involved in the MET signaling pathway, are displayed in Figure 4a.

### 3.8. Higher SOX2 Protein Expression in the UWB1.289 Cell Line with Restored BRCA1

Western blot analyses for the presence of MET, the stem cell factors SOX2, Nanog, and OCT4, as well as the HR markers RAD51 and BRCA1 were performed for the UWB1.289 and UWB1.289+BRCA1 cell lines, as mRNA data for the *BRCA1*-proficient cell line were not available in the CCLE database. Results are displayed in Figure 4b and Supplementary Figure S6. As expected, BRCA1 was only detected in the proficient line. MET, RAD51, and OCT4 levels were comparable in the two lines; however, SOX2 was considerably higher in the *BRCA1*-proficient cell line. Nanog levels were below the level of detection by Western blot in both lines.

## 4. Discussion

*MET* was discovered to be a potent oncogene in the 1990s when studies reported that when co-expressed with its ligand, hepatocyte growth factor (HGF), it promoted metastases in an animal model [35]. Overexpression has since been linked to tumor development in mouse models, and activating mutations have been found in renal and non-small-cell lung cancers [9]. MET and its ligand HGF have also been shown to interact with Wnt signaling to maintain stemness in colon cancer cells [36]. The prognostic role has also been evaluated, revealing e.g., shorter OS for patients with MET-positive ovarian tumors [10]. We set out to investigate the connection between MET and stemness in HGSC and to evaluate the prognostic role of this oncogene in a consecutive cohort of HGSC, alone and in combination with stem cell markers. We found MET to be a negative predictor of OS and PFS, but more strikingly, we discovered that this effect was most evident in tumors negative for SOX2 expression. To explain this connection, we hypothesized that tumor cells with a stem cell phenotype are less dependent on MET signaling, but in tumors lacking the stem cell factor network where SOX2 is included, signaling through MET may be more crucial for cancer progression. We further studied a large TCGA data set in order to validate these findings. These data are of bulk mRNA expression from microarrays, and we used the cut-off of z-score > 0 to define positive expression. This means that results cannot be fully compared to the more informative TMA scoring, but trends could still be detected. *MET* did not have an effect on survival in the SOX2-negative subgroup; however, in the *POU5F1*-negative subgroup, we saw similar effects as in the TMA cohort. The SOX2 and OCT4 proteins interact closely by forming dimers with the ability to bind specifically to genes involved in cell fate and pluripotency [37]. Both could potentially be connected to MET, considering that high MET expression has been previously associated with cancer stem cell subpopulations in glioblastoma [25,38]. The discordance between our IHC data and the TCGA mRNA data may be explained by issues with probe quality in the microarray and by the fact that bulk mRNA analysis is less sensitive for the detection of minor cell populations. Combining the four closely connected factors *SOX2*, *POU5F1*, *Nanog,* and *KLF4*, we observed that *MET* expression affected OS in patients whose tumors expressed none or only one of the factors. This indicates that these factors may complement each other and suggests that the tumor relies on other mechanisms for progression when an insufficient number of stem cell factors are present. Further studies are required to confirm these theories. In this context, Robinson et al. recently reported a stronger role for *SOX2* compared to *OCT4* or *Nanog* for tumor relapse potential following chemotherapy in ovarian cancer patients and that HGSC cell lines displayed greater chemoresistance and increased expression of *SOX2*, *OCT4*, and *Nanog* when grown in 3D conditions compared to 2D [39].

To explore potential links between PARPi response, copy number aberrations, and cancer stem cell factors, we compared the copy number landscape and HRD scores in a cohort consisting of patients receiving either olaparib maintenance or surveillance (Cohort 2). We found, surprisingly, small differences between the groups with and without *BRCA1/2* mutations. This may be due to generally high HRD scores in this cohort, since all patients were platinum-sensitive and had at least a partial response to re-treatment with platinum. The HRD scores for the whole cohort were nonetheless comparable to the range of scores previously reported in HGSC [40]. Using the cut-off HRD > 42 established in breast cancer [31], 84% of the patients were HRD-positive. PFS was comparable between the groups, but patients with *BRCA1/2* mutations had a longer OS, which could be expected as they received olaparib treatment and, as a group, also generally respond better to platinum treatment [41].

Amplification of *SOX2* was common, as has previously been reported in several cancer forms, including HGSC [29]. The commonly amplified region on the 3q arm, however, comprises many other potential driver genes (e.g., *MECOM*, *PIK3CA, ECT2*, and *PRKCI*). Whether *SOX2* should be considered a passenger or a driver gene is not fully determined. In this study, SOX2 protein levels appeared to correlate with *SOX2* amplification, supporting the notion that it plays a role in cancer development and that overexpression of SOX2 to some extent is dependent on amplification.

Comparing growth inhibition in cell line pairs, we found that the post-treatment platinum-resistant PEO4 cells were more sensitive to olaparib treatment than PEO1 cells, although PEO1 cells harbor a *BRCA2* mutation, and PEO4 has a reversal mutation restoring *BRCA2* function. This result contradicts earlier reports, which stated that the PEO1 line was more sensitive [33,42]. We find that comparing cell lines is challenging, as growth patterns differ and exponential growth phases are reached at different time points in these two cell lines (see growth curves deposited at phe-culturecollections.org.uk). Results are therefore dependent on the incubation time used, and this differed in our studies. Therefore, no firm conclusion can be drawn on the true difference in response to PARPi between these cell lines. This may also reflect the considerable heterogeneity in treatment response observed among HGSC patients as well the intra-tumor genetic heterogeneity [42]. We evaluated mRNA data for these cell lines to explore whether we could find a possible explanation for the observed inverse response to PARPi compared to carboplatin. We found that PEO4 cells expressed lower levels of genes involved in HR function compared to PEO1 cells, which could explain why they responded strongly to PARPi, despite the restored *BRCA2* function. PEO1 also expressed higher levels of several key stem cell factors, which could indicate a more stem cell-like phenotype. As cancer stem cells have been reported to express higher levels of drug transporter pumps, have a more intact DNA repair system, and are more prone to epithelial-to-mesenchymal transition (EMT) [43], a higher tolerance to PARPi seems plausible for cells expressing higher levels of stem cell factors. It has been reported that platinum-sensitive tumors respond better to PARPi, with a response rate of 61.5% for platinum-sensitive disease compared to 41.7% for platinum-resistant and 15.4% for platinum-refractory patients [44], and approval for PARPi has therefore primarily focused on the platinum-sensitive patient group. Nevertheless, response rates are relatively high among patients with acquired resistance—PEO4 cells could be categorized as such—and the PARPi sensitivity we observed is therefore not surprising.

In order to compare gene activity in the UWB1.289 and UWB1.289+BRCA1 cell lines, Western blot analyses were performed on selected proteins, as the UWB1.289+BRCA1 cell line was not included in the CCLE DepMap mRNA data set. The Western blot analyses revealed markedly higher protein expression of SOX2 in the *BRCA1*-proficient line compared to the *BRCA1*-deficient line. This may indicate that the platinum and PARPi resistance acquired through the restoration of the *BRCA1* function is connected to the activation of stem cell networks. Of note, the recently concluded PRIMA trial reported benefit in all patient groups receiving niraparib as first-line maintenance treatment, provided complete or partial response to upfront platinum therapy irrespective of the HRD status [45], clearly indicating that the currently used predictive biomarkers need to be improved for better patient selection and that clinical guidelines pertaining to PARPi are subject to change as novel trial data and biological understanding emerge.

Synergistic effects of olaparib and the c-MET inhibitor crizotinib have been reported in earlier studies, including ours [15,16,17], but the benefit of combining PARPi and METi could not be verified in the cell lines representing treatment resistance used in the present study. Crizotinib also inhibits ALK and ROS1, in addition to MET, but possible effects on downstream signaling in these pathways were not further explored in this study, as all four cell lines lacked the expression of ALK and ROS1.

## 5. Conclusions

We conclude that MET expression could be used as a prognostic tool in HGSC, but measures of stemness should be taken into consideration when further evaluating the mechanism/s underlying the driving effect of this receptor tyrosine kinase in cancer. Combining MET and PARP inhibitors did not increase cancer cell death in vitro in this study; nevertheless, MET inhibition could still be a viable treatment option. It should, however, be noted that patients with tumors expressing stem cell factors might not benefit from the treatment. Including stem cell marker screening in future clinical trials could help pinpoint the subgroup of patients eligible for METi treatment.

## Figures and Tables

**Table 1:** Clinico-pathological variables in relation to MET expression.

	All (n = 130)	MET+ (n = 31)	MET- (n = 99)	*p*
***Age, median (range)***	67 (43–86)	66 (43–85)	66 (45–86)	0.71 ^T^
***BRCA1/2 status***				1.0 ^F^
*Mutant*	16 (36)	3 (38)	13 (35)	
*Wildtype*	29 (64)	5 (63)	24 (65)	
*NA*	85	23	62	
***Residual disease, n (%)*^1^**				1.0 ^F^
*No*	76 (58)	18 (58)	58 (59)	
*Yes*	54 (41)	13 (42)	41 (41)	
***Treatment response, n (%)***				0.067 ^W^
*Complete response*	86 (72)	18 (60)	68 (76)	
*Partial response*	30 (25)	10 (33)	20 (22)	
*Progressive disease*	3 (2.5)	2 (6.7)	1 (1.1)	
*Undetermined*	11	1	10	
***Platinum sensitivity***				0.23 ^F^
*≥* *6 months*	93 (75)	20 (66)	73 (76)	
*<6 months*	31 (25)	10 (33)	21 (22)	
*NA*	6	1	5	
***Stage, n (%)***				0.086 ^F^
*III*	100 (77)	20 (65)	80 (81)	
*IV*	30 (23)	11 (36)	19 (19)	
***Chemotherapy, n (%)***				0.015 ^F^
*Carboplatin combination*	111 (85)	24 (77)	87 (88)	
*Carboplatin single*	12 (9.2)	4(13)	8 (8.0)	
*Other*	3 (2.3)	3 (9.7)	0 (0)	
*No chemo*	4 (3.1)	0 (0)	4 (4.0)	

^1^ Macroscopic disease remaining after primary debulking surgery; ^T^ Student’s two sample *t*-test; ^F^ Fisher’s exact test; ^W^ Wilcoxon rank–sum test.

## Data Availability

The data presented in this study are available on request from the corresponding author. The data are not publicly available due to GDPR restrictions.

## Supplementary Materials

The following are available online at https://www.mdpi.com/article/10.3390/genes12050742/s1, Figure S1: Representative images of MET IHC; Figure S2: Survival outcome for different levels of MET; Figure S3: Correlations between MET and stem cell factors and prognostic value of MET in stem cell factor-negative tumors; Table S1: Clinico-pathological data, Cohort 2; Figure S4: Relative gain (total copy number/ploidy) in 40 core HR-associated genes; Figure S5: Frequency plots of global copy number alterations in cohort 2; Figure S6: Quantification of Western blot analyses.
