# Supplementary material for: MET Expression and Cancer Stem Cell Networks Impact Outcome in High-Grade Serous Ovarian Cancer
Source: Genes (Basel). 2021 May 14;12(5):742. doi: 10.3390/genes12050742 (PMC8155853; doi:10.3390/genes12050742)
Supplement: Supplementary file 1 [file genes-12-00742-s001.zip › genes-1202950-supplementary.pdf]

Supplementary material

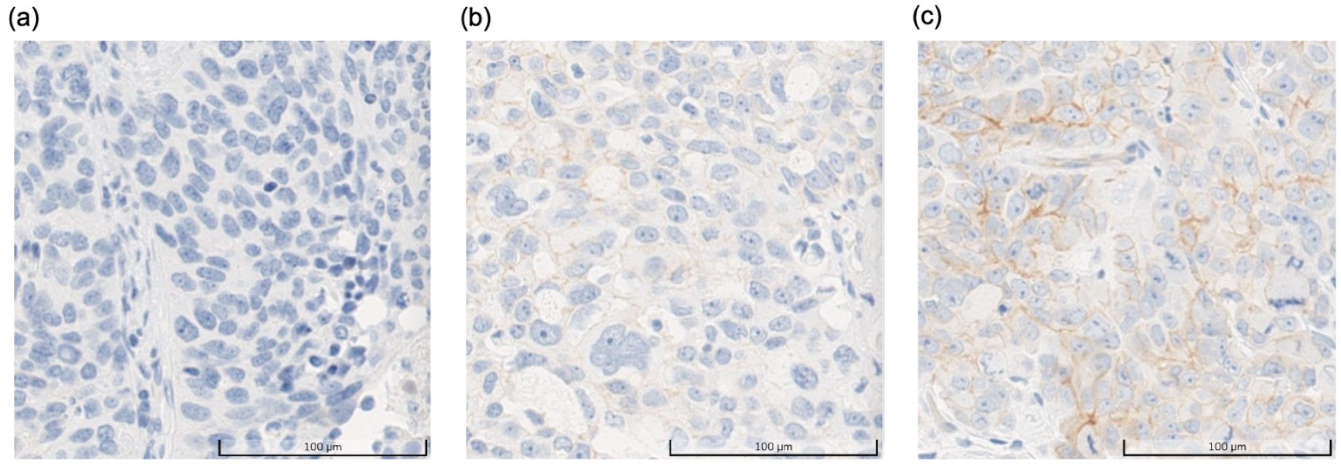

Supplementary Figure 1. Representative images of MET IHC.

TMA core negative for membranous staining (a), membranous staining of weak intensity (b) and membranous staining of moderate intensity (c). Cores were scored as MET positive if ≥5% cells had weak or moderate membrane staining. Magnification 20x.

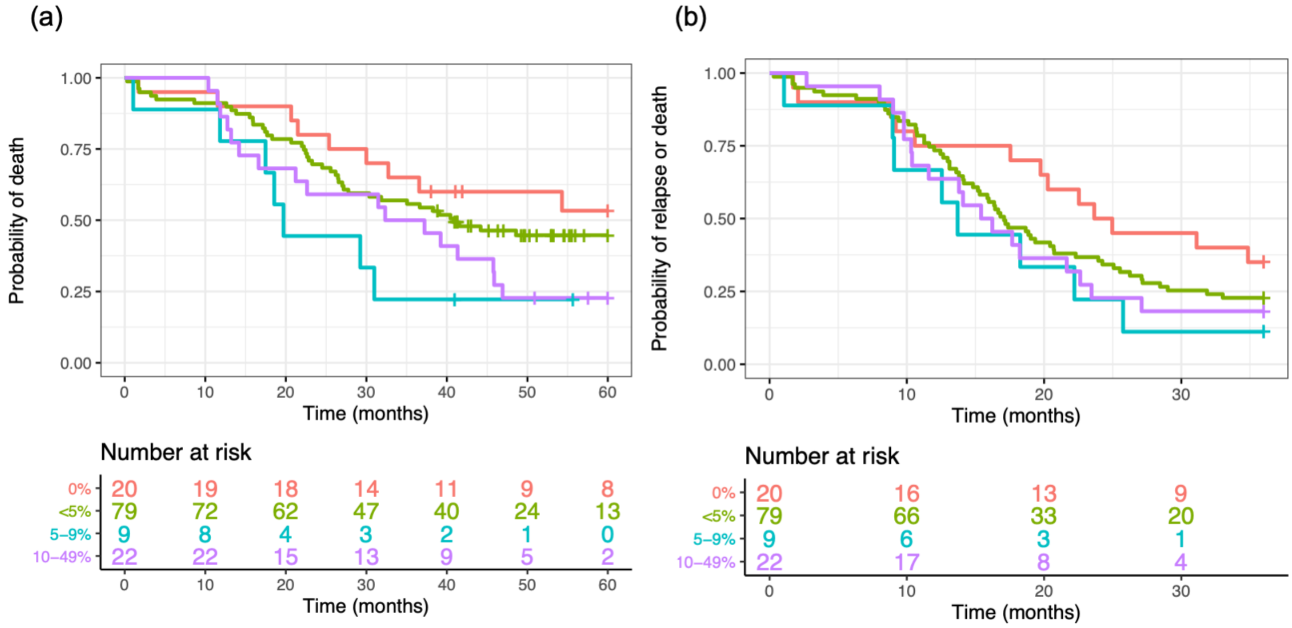

Supplementary Figure 2. **Survival outcome for different levels of MET**; OS (a) and PFS (b).

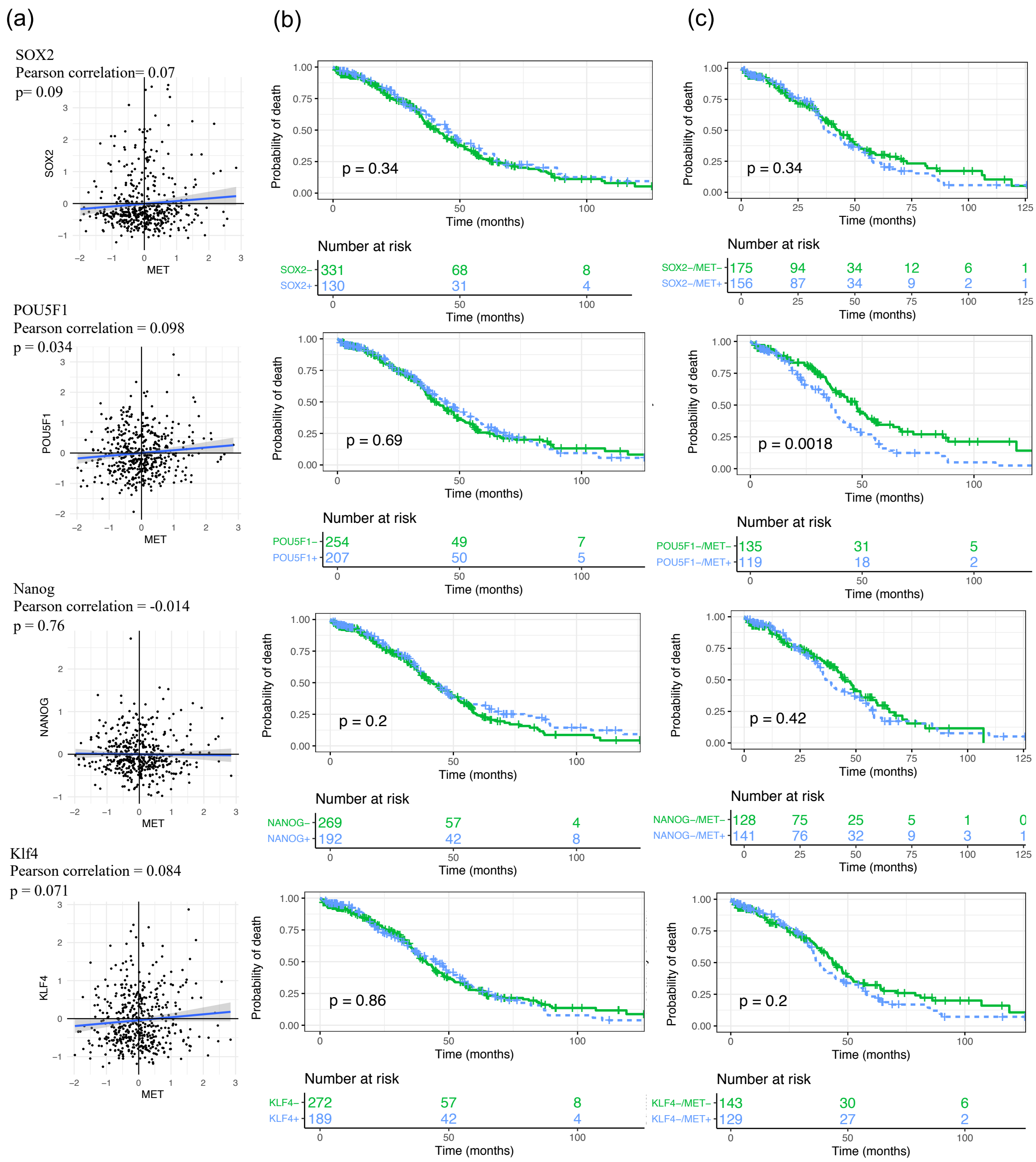

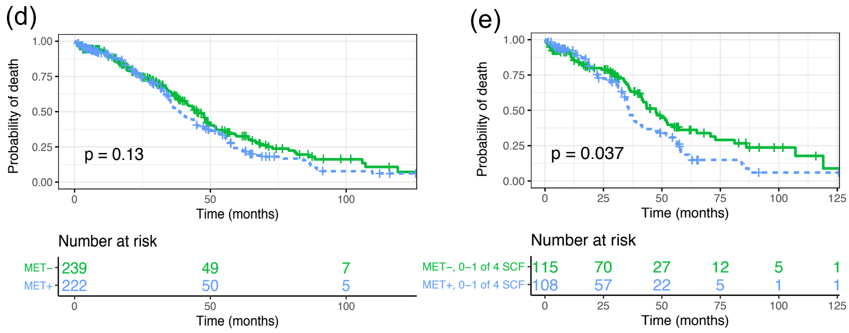

(f)

Stem cell factor	Tumors (n)
SOX2	21
POU5F1	46
Nanog	37
KLF4	44
No gene expressed	75

Supplementary Figure 3. Correlations between MET and stem cell factors, and prognostic value of MET in stem cell factor negative tumors. Pearson correlation of mRNA levels of four stem cell factors (and MET (a). Kaplan-Meier analyses of OS for the four stem cell factors (b). Subgroup analyses of OS for MET+/- in tumors negative for the respective stem cell factors (c). OS for MET+/- in all tumors (d) and in tumors negative for at least three out of the four stem cell factors (e). The number of tumors positive for each stem cell factor in the subgroup analysis (f).

Supplementary Table 1. Clinico-pathological data, cohort 2

	All (n=25)	*BRCA1/2 mutated (n=10)*	*BRCA1/2 wildtype (n=15)*
Age, median (range)[1]	60 (42-55)	59 (42-66)	61 (43-78)
Residual disease, n (%)[2]			
No	21 (84)	6 (60)	15 (100)
Yes	4 (16)	4 (40)	0 (0)
FIGO stage, n (%)			
I-II	2 (8.0)	2 (20)	0 (0)
III	18 (72)	5 (50)	13 (87)
IV	5 (20)	3 (30)	2 (13)
Treatment response, n (%)			
Complete response	23 (92)	9 (90)	14 (93)
Partial response	2 (8.0)	1 (10)	1 (7)
Deceased, n (%)	15 (63)	5 (50)	10 (71)
Overall survival, months, median (range)[3]	63 (23-146)	64 (31-119)	49 (23-71)
Progression-free survival, months, median (range)[4]	21 (14-58)	21 (14-58)	20 (14-43)
HRD score, median (range)	57 (26-85)	63.5 (38-85)	52 (26-76)

[1]Age at initial diagnosis. [2]Tumor tissue remaining after primary debulking surgery. [3]Only deceased patients included in OS calculations. [4]Time to first relapse, before olaparib treatment.

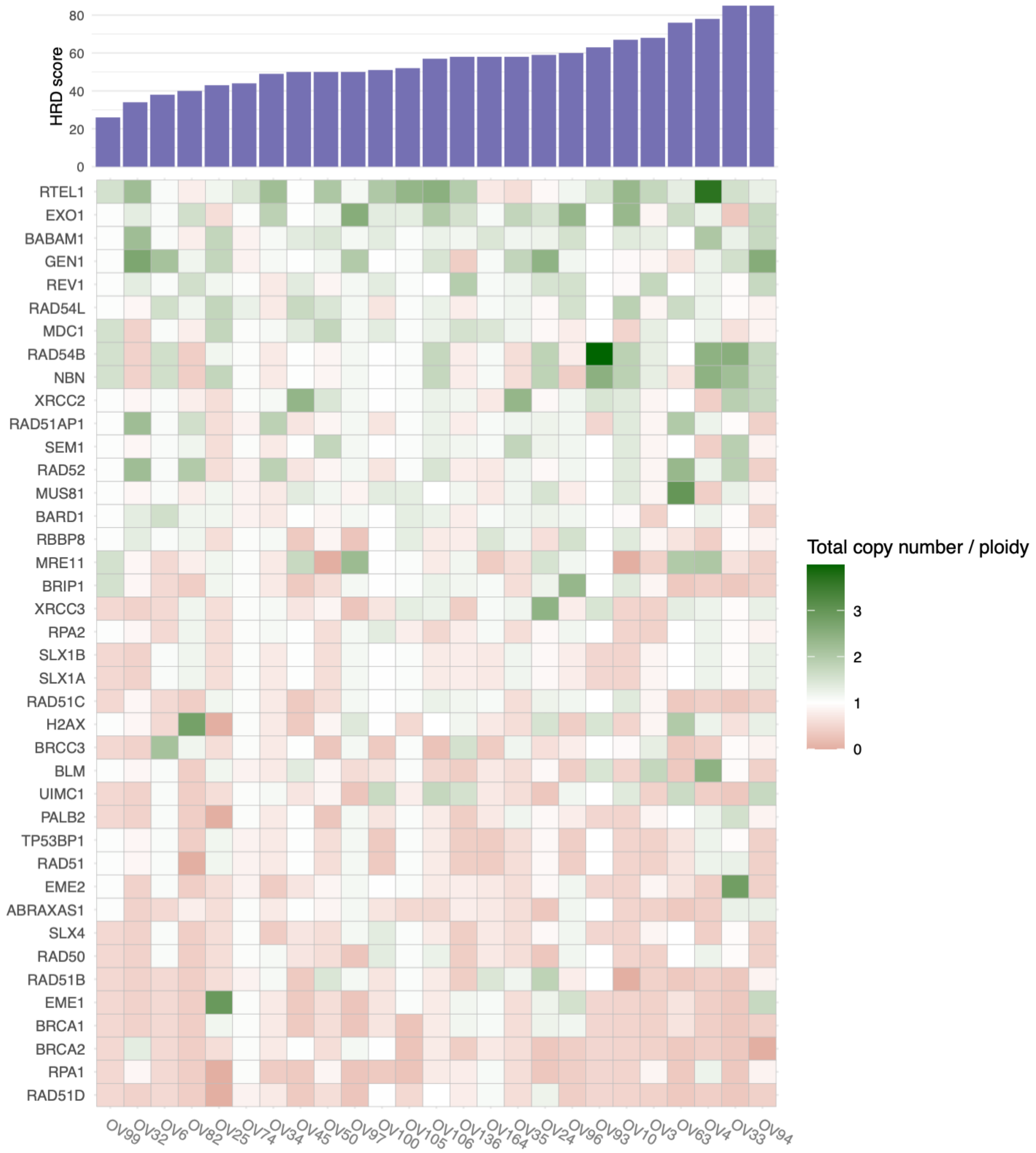

Supplementary Figure 4. **Relative gain (total copy number/ploidy) in 40 core HR-associated genes**.

Samples sorted by HRD score (x-axis) and genes sorted by mean relative gain (y-axis).

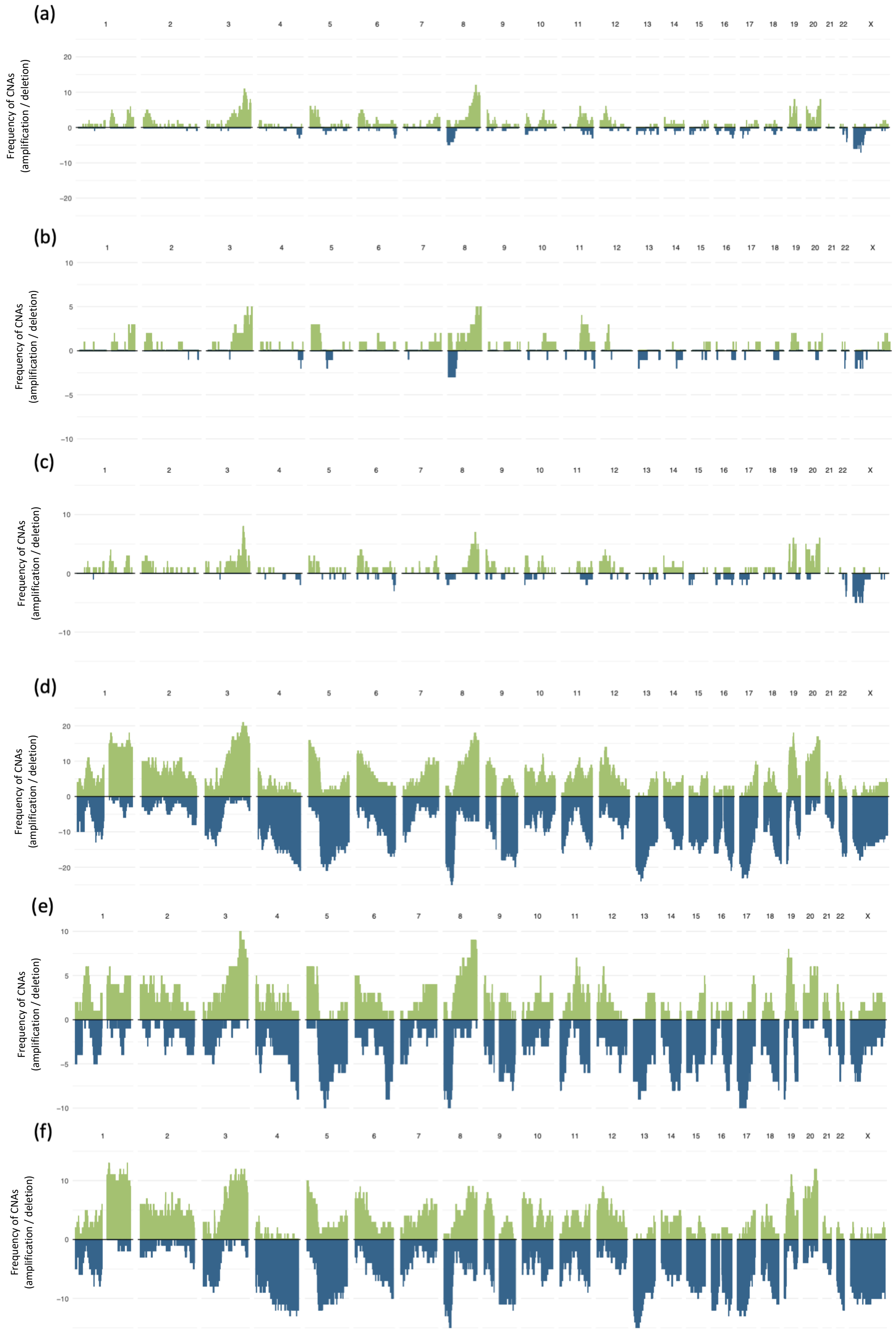

Supplementary Figure 5. Frequency plots of global copy number alterations (CNAs) in cohort 2. Amplification/Deletion (a-c) and Gain/Loss (d-f). (a) & (d): all patients (n=25), (b) & (e): patients with a *BRCA1/2* mutation (n=10) and (c) & (f): patients with wildtype *BRCA1/2* (n=15).

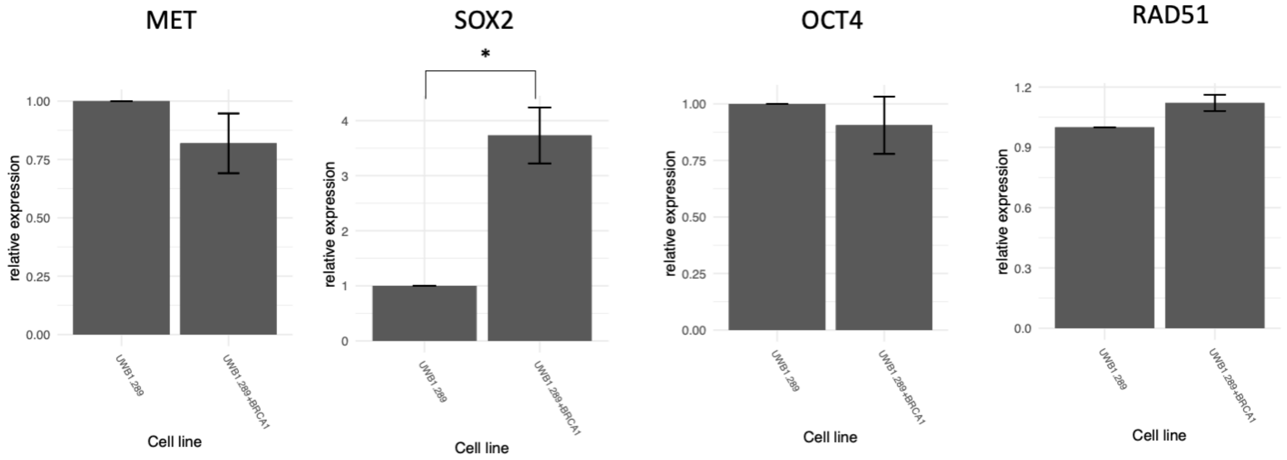

Supplementary Figure 6. Quantification of Western blot analyses. Analyses were performed with three biological replicates. Means and SE displayed. Relative protein expression for BRCA1 could not be calculated as the protein was not detected in the UWB1.289 cell line.

* significant Student's T-test, p=0.033
